# The pathophysiological mechanisms of immunosenescence in coronary artery disease

**DOI:** 10.3389/fcell.2025.1686947

**Published:** 2025-10-08

**Authors:** Hengjie Bie, Zhengxian Tao

**Affiliations:** Department of Cardiology, The First Affiliated Hospital of Nanjing Medical University, Nanjing, Jiangsu, China

**Keywords:** immunosenescence, coronary artery disease, atherosclerosis, inflammaging, senescence-associated secretory phenotype (SASP)

## Abstract

Coronary artery disease (CAD) is the most common coronary heart disease, characterized by the accumulation of atherosclerotic plaques in the coronary arteries, which supply oxygen and nutrients to the heart. The National Health and Nutrition Examination Survey (NHANES) reported that between 2011 and 2014, the prevalence of coronary artery disease was higher in men (30.6%) than in women (21.7%) aged ≥80 years. In the ARIC (Atherosclerosis Risk in Communities) study, the incidence of myocardial infarction (MI) was higher in black individuals compared to white individuals among those aged 65–84 years. Immunosenescence plays a pivotal role in its onset and progression. Immunosenescence is a complex process involving organ remodeling and cellular regulation, leading to a decline in immune function and reduced responses to infection and vaccination in older adults. By driving dysfunction in multiple immune cell populations—including T cells, B cells, and macrophages—immunosenescence promotes chronic inflammation, vascular injury, and the advancement of atherosclerotic plaques. In recent years, intervention strategies targeting immunosenescence—such as restoration of hematopoietic stem cell function, reconstitution of T- and B-cell compartments, modulation of macrophage polarization and effector programs, and thymic regeneration—have made substantive progress. Future research should prioritize elucidating the mechanisms of immunosenescence, advancing the development of personalized therapeutic strategies, and rigorously validating their efficacy and safety in clinical trials; therapeutic modulation of immunosenescence holds promise for improving treatment outcomes and prognosis in patients with CAD.

## 1 Introduction

Coronary artery disease (CAD) is the most common type of coronary heart disease, characterized by the accumulation of atherosclerotic plaques in the coronary arteries, which supply oxygen and nutrients to the heart (Tsao et al., 2022). It is a leading cause of mortality and disability, with approximately 380,000 deaths in 2020. The NHANES reported a higher prevalence of CAD in men (30.6%) than in women (21.7%) aged ≥80 years between 2011 and 2014 (Benjamin et al., 2017; National Heart and Institute, 2006; Global, 2015). Similar findings were observed in the FHS (Framingham Heart Study) and CHS (Cardiovascular Health Study). In the ARIC (Atherosclerosis Risk in Communities) study, the incidence of myocardial infarction (MI) was higher in black individuals compared to white individuals among those aged 65–84 years. CAD is a chronic inflammatory disorder, with atherosclerosis as the central pathological process. Clinically, it presents as stable angina, unstable angina, myocardial infarction (MI), or sudden cardiac death (Ross, 1999; Álvarez-Álvarez et al., 2017). Atherosclerosis involves the formation of plaques in the vascular intima, primarily driven by chronic inflammation due to various risk factors, including cholesterol accumulation and the retention of lipoproteins such as low-density lipoprotein (LDL) in the arterial walls (Basatemur et al., 2019; Soehnlein and Libby, 2021).

The assessment of CAD can be categorized into anatomical and functional approaches (de Oliveira Laterza Ribeiro et al., 2023). Anatomical evaluation is primarily performed using coronary angiography and coronary computed tomography (CCT), the latter offering advantages in noninvasive imaging, vascular structural analysis, and prediction through coronary artery calcium (CAC) scoring. However, anatomical methods have limitations in determining the functional significance of coronary lesions; therefore, functional assessment serves as an essential complement. Commonly used functional techniques include exercise electrocardiography (ECG), stress echocardiography, single-photon emission computed tomography (SPECT), cardiac magnetic resonance imaging (CMR), and positron emission tomography (PET), with these imaging modalities demonstrating diagnostic accuracies exceeding 80% (Takx et al., 2015). The primary objectives of CAD treatment are to alleviate symptoms and prevent complications in patients with stable CAD, while for those with acute coronary syndrome (ACS), the goal is to improve coronary blood flow and restore cardiac function as rapidly and effectively as possible (Ford et al., 2018; Herrmann et al., 2012). Traditionally, these goals are achieved through pharmacological therapy, with consideration of percutaneous coronary intervention (PCI) or coronary artery bypass grafting (CABG) when necessary. For patients with stable CAD, pharmacological management typically includes beta-blockers, calcium channel blockers, nitrates, angiotensin-converting enzyme inhibitors (ACEIs), and statins, whereas for patients with ACS, treatment primarily involves thrombolytic agents and antiplatelet therapies (Kh et al., 2017). In recent years, beyond traditional risk factors (Yusuf et al., 2004)—including hyperlipidemia, hypertension, diabetes mellitus, and cigarette smoking—alterations in immune system function (Jonasson et al., 2003), particularly immunosenescence (Santoro et al., 2021; Liu et al., 2023), have been recognized as critical drivers of the onset and progression of CAD.

Immunosenescence is a complex process involving organ remodeling and multilayered cellular regulation, characterized by a progressive decline in immune competence that compromises responses to infections and vaccinations in older adults (Nikolich-Žugich, 2018; Lanna et al., 2017; Ucar et al., 2017). Key features include thymic involution, hematopoietic stem cell (HSC) dysfunction, altered naïve-to-memory T- and B-cell ratios, chronic low-grade inflammation (“inflammaging”), senescent cell accumulation, impaired recognition of novel antigens, mitochondrial dysfunction, metabolic dysregulation, and increased genomic instability. With advancing thymic atrophy (Goronzy and Weyand, 2013), functional epithelial regions are gradually replaced by non-thymopoietic perivascular spaces, resulting in reduced peripheral naïve T cells, expansion of terminally differentiated memory T cells, and restricted T-cell egress.

Adults thymectomized in early childhood exhibit premature immunosenescent profiles. Nevertheless, thymic degeneration alone does not fully account for diminished T-cell receptor (TCR) diversity, and thymic rejuvenation may not restore it. Immunosenescence arises from the persistent accumulation of endogenous damage, where senescent cells sustain low-grade chronic inflammation through the secretion of a senescence-associated secretory phenotype (SASP), which includes IL-1, IL-6, IL-8, IL-13, IL-18, TNF, and their corresponding receptors. Cellular senescence exemplifies pleiotropy—providing early-life benefits by suppressing tumorigenesis and promoting tissue development and repair, but exerting detrimental effects in later life (Feldman et al., 2015). Metabolic reprogramming accompanying immunosenescence—characterized by increased glycolysis, mitochondrial dysfunction, and elevated reactive oxygen species—closely associates with the high late-life incidence of cardiovascular, neurodegenerative, autoimmune, and metabolic diseases, as well as cancer. This review summarizes the mechanistic roles of immunosenescence in CAD and discusses its therapeutic potential, with the aim of informing new strategies for the prevention and treatment of CAD.

## 2 Immunosenescence and CAD

### 2.1 T cell

As the immune system ages, the balance of immune cell populations shifts, characterized by a decline in naïve T cells and an expansion of memory and senescent T cells (Nikolich-Žugich, 2018; Ghamar Talepoor and Doroudchi, 2022). Senescent T cells typically lose CD28 and acquire CD57 expression; these cells are terminally differentiated, exhibit potent pro-inflammatory and cytotoxic activity, and are resistant to apoptosis and regulation by regulatory T cells (Tregs) (Vallejo et al., 1999; Formentini et al., 2021; Álvarez-Heredia et al., 2023; Broadley et al., 2017). In addition to reductions in proliferative naïve T and B cells with a relative increase in central memory subsets, aging is accompanied by contraction of the T-cell receptor (TCR) repertoire, resulting in diminished antigen responsiveness. Studies have shown that CD28^−^CD57^+^ T cells can directly or indirectly kill endothelial cells (Namekawa et al., 2000), thereby contributing to vascular injury. Expansion of CD28^−^CD57^+^ T cells is associated with heightened inflammatory responses and increased oxidative stress, both of which promote atherogenesis and the pathogenesis of CAD (Téo et al., 2013; Dumitriu et al., 2009). Consequently, this phenotypic shift in T cells, together with other age-related immune alterations, sustains a chronic inflammatory milieu that drives the initiation and progression of CAD (Bergström et al., 2012).

### 2.2 B cell

B cells exert bidirectional regulation in atherosclerosis. B2 cells constitute the predominant B-cell pool in the circulation and secondary lymphoid organs; among them, follicular B cells (FO B cells) are pro-atherogenic (Snijckers and Foks, 2024). Through cooperation with T follicular helper (Tfh) cells, FO B cells undergo activation and enter germinal centers, differentiating into GC B cells that produce high-affinity IgG (Sage et al., 2019) autoantibodies against oxidation-specific epitopes (OSEs)—including stress-induced endothelial HSP60/65, malondialdehyde (MDA) and 4-hydroxynonenal (4-HNE) generated from oxLDL, and phosphocholine-containing oxidized phospholipids. These IgG antibodies increase under hyperlipidemic conditions, amplifying inflammation and enlarging plaques. Experimentally, engagement of the BTLA pathway reduces FO B cells by ∼50% and significantly decreases plaque burden; FO deficiency induced by targeted deletion of Blimp-1 or Pax5 similarly lowers IgG levels and attenuates lesions. Conversely, transfer of IgG from atherosclerotic mice into Ldlr−/− mice lacking endogenous IgG aggravates plaque development. In contrast to FO B cells, marginal zone (MZ) B cells suppress the atherogenic functions of Tfh cells and, in a T-cell–dependent manner, produce atheroprotective IgM. B1a/B1b cells likewise confer protection by secreting large amounts of germline-encoded natural IgM recognizing OSEs, thereby promoting non-immunogenic clearance of oxLDL and apoptotic cells (Centa et al., 2018; Binder et al., 2016). IL-10^+^ regulatory B cells inversely correlate with plaque severity, although (Tsiantoulas et al., 2012) B-cell–specific IL-10 deficiency does not alter lesion size, suggesting that their protective effects may be partly—or largely—independent of IL-10 (Douna et al., 2019).Overall, the impact of B cells on atherogenesis is determined by subset identity, crosstalk with Tfh cells, and antibody isotype: IgG generally promotes plaque progression, whereas IgM primarily mediates neutralization and clearance (Sage et al., 2015).

### 2.3 Macrophage

The changes in macrophages during immunosenescence represent a complex process driven by multiple factors, centered on the interplay between functional impairment and the acquisition of cellular senescence-associated characteristics (Sharma, 2021). Macrophages span both the initiation and the resolution phases of inflammation, and their M1 (pro-inflammatory) versus M2 (pro-repair) polarization balance shifts with age in a tissue- and context-dependent manner: a bias toward M2 has been reported in aging skeletal muscle and in macular degeneration, whereas a shift toward M1 has been attributed to enteric nervous system degeneration in aged mice (Mahbub et al., 2012; Wynn et al., 2013; Cui et al., 2019; Zandi et al., 2015; Sharma et al., 2014). Regardless of direction, any imbalance disrupts host defense and resolution programs, rendering older individuals more susceptible to infections and chronic inflammation. Functionally, aged macrophages display reduced phagocytosis and respiratory burst (Chelvarajan et al., 2006; Linehan et al., 2014; Wong et al., 2017; Swift et al., 2001), diminished TLR expression (Davila et al., 1990; Herrero et al., 2001), blunted responses to antigenic stimulation, and lowered LPS-induced cytokine production (Ding et al., 1994). Notably, bone-marrow–derived macrophages do not fully recapitulate these defects, indicating that age-associated dysfunction is not purely cell-intrinsic but is strongly shaped by extrinsic microenvironmental cues. Antigen presentation is likewise compromised: after IFN-γ stimulation, induction of MHC class II and the capacity to activate T cells are reduced, slowing and weakening adaptive immunity. In tissue repair, aging alters chemokine milieus and patterns of macrophage infiltration, delays efferocytosis, and thereby impairs wound healing. Following vascular injury, aged arteries form thicker neointimas, correlating with increased tissue macrophages and elevated IL-18; depletion of macrophages mitigates this phenotype.

From the perspective of cellular senescence, macrophages in old age or in DNA-repair–deficient settings upregulate p16INK4a and p21CIP1, acquire a SASP, adopt elongated morphologies, and exhibit SA-β-gal activity—features consistent with their serving as *in vivo* sources of SASP that amplify inflammation. That said, some evidence suggests p16/SA-β-gal expression in macrophages can reflect reversible activation states rather than *bona fide* senescence, underscoring the need to distinguish these conditions rigorously. Telomere biology provides a more direct etiologic thread: age-related telomere attrition or telomerase loss drives oxidative stress, mitochondrial abnormalities, and hyperactivation of the NLRP3 inflammasome, thereby impairing phagocytosis, GM-CSF–dependent proliferation, and signaling (e.g., Stat5a oxidation/phosphorylation). These changes align with inflammatory phenotypes observed in chronic diseases such as type 2 diabetes and sickle cell disease. Accumulating ROS both drives senescence and reinforces immune dysfunction, creating a feed-forward loop (Frodermann and Nahrendorf, 2018; Frangogiannis, 2021).

In summary, macrophage immunosenescence is sculpted by intertwined intrinsic factors (telomere/mitochondrial damage, cell-cycle inhibition, SASP) and extrinsic factors (aging tissue microenvironments and low-grade sterile inflammation). The result is a coexistence of effector hypofunction with dysregulated inflammatory control. Although definitive causal chains linking cellular senescence and immunosenescence in macrophages remain to be established, current evidence nominates actionable targets focused on reducing oxidative stress, rebalancing M1/M2 polarization, restoring antigen presentation, and constraining the SASP.

### 2.4 Inflammaging

“Inflammaging” refers to the chronic, low-grade inflammatory state that emerges during aging (Franceschi et al., 2018). It is primarily macrophage-centered and characterized by a complex balance between pro-inflammatory and anti-inflammatory responses. According to current literature, the major sources of inflammatory stimuli are endogenous, mislocalized, or structurally altered molecules derived from damaged or dying cells and organelles (cellular debris), which are recognized by receptors of the innate immune system. Although the production of these molecules is physiological and increases with age, their clearance through autophagy, mitophagy, and proteasomal degradation progressively declines. This “autoreactive/autoimmune” process drives the onset and progression of chronic diseases and can accelerate and amplify aging both locally and systemically.

**FIGURE 1:**
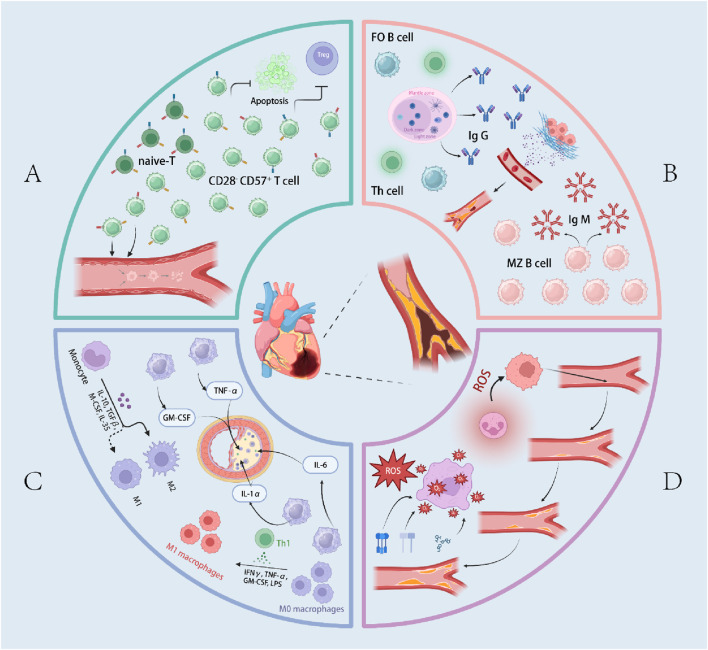
Overview of immunosenescence in Coronary artery disease (CAD). **(A)** represents the pathogenic mechanisms of T cells in CAD within the context of immunosenescence. **(B)** illustrates the pathogenic mechanisms of B cells in CAD within the context of immunosenescence. **(C)** depicts the pathogenic mechanisms of macrophages in CAD within the context of immunosenescence. **(D)** outlines the pathogenic mechanisms of inflammaging in CAD. The terms “follicular B cells (FO B cells)”, “marginal zone B cells (MZ B cells)”, and “reactive oxygen species (ROS” are also included.

“Inflammaging” is a major risk factor for cardiovascular disease (CVD), characterized by persistently elevated pro-inflammatory cytokines that drive endothelial injury, impaired vascular remodeling, and atherosclerosis (Franceschi et al., 2018; Donato et al., 2015). These cytokines arise predominantly from senescent T cells and pro-inflammatory macrophages (Libby et al., 2009), reflecting age-related failures in immune regulation that disrupt tissue homeostasis (Leon and Zuckerman, 2005; Franceschi and Campisi, 2014). During cardiac stress, ischemic injury, or metabolic syndrome, necrotic cells release damage-associated molecular patterns (DAMPs) that are sensed by pattern-recognition receptors (PRRs) on innate immune cells, triggering robust inflammation (Mauro et al., 2019). The ensuing secretion of pro-atherogenic cytokines, reactive oxygen species (ROS), and reactive nitrogen species (RNS) amplifies oxidative stress, promotes vascular smooth muscle cell (VSMC) proliferation, and fosters the accumulation of oxidized low-density lipoprotein (LDL), which is taken up by macrophages to form foam cells and destabilize plaques. On the T-cell axis, expansion of senescence-associated cytotoxic populations—particularly CD8^+^CD28^−^ T cells—is closely linked to vascular dysfunction, while late-differentiated CD4^+^CD28^−^ T cells that produce IFN-γ increase in unstable angina. At the population level, cytomegalovirus (CMV)–associated atherosclerosis in older men may be mediated by a higher proportion of memory CD4^+^ T cells. Collectively, an aging immune system, through DAMP–PRR–driven inflammatory amplification and T-cell repertoire remodeling, orchestrates endothelial damage, oxidative stress, lipid deposition, and maladaptive vascular remodeling, forming the immunologic substrate for CVD onset and progression. The above-mentioned pathogenesis is shown in Figure 1.

## 3 Therapeutic strategy

### 3.1 Immune progenitors (hematopoietic stem cells)

Anti-aging interventions for hematopoietic stem cells (HSCs) can be implemented as an integrated program of “damage mitigation, metabolic stabilization, telomere protection, polarity correction, and source replacement.” First, to counter DNA damage and elevated reactive oxygen species (ROS) (Brown et al., 2013) in aged HSCs, inhibition of the p38 stress pathway (Hsieh and Papaconstantinou, 2002; Li et al., 2011) together with enhancement of antioxidant defenses can lower oxidative stress and improve self-renewal and hematopoietic potential; in parallel, restoring mitochondrial homeostasis (e.g., via upregulation of Sirt3) and re-establishing autophagy—particularly reactivation of chaperone-mediated autophagy (Dong et al., 2021) (CMA)—helps correct energetic imbalance and bolster hematopoietic resilience. Second, preservation of telomere integrity—within strict safety boundaries—through telomerase activation/gene therapy or reinforcement of telomere-binding proteins may alleviate telomere-driven DNA damage responses and myeloid skewing (Allsopp et al., 2003), while necessitating careful stratification given potential oncogenicity. Third, correcting cell polarity and lineage bias by inhibiting hyperactive cdc42 (Hosokawa et al., 2017; Hammad and Lambrecht, 2008) (e.g., with the small molecule CASIN) can restore HSC polarity, enhance lymphoid progenitor output, and re-establish immune homeostasis, with evidence for broader rejuvenation across stem-cell compartments. Fourth (Kovina et al., 2019), “source replacement” via transplantation of bone marrow/hematopoietic grafts from young donors (Das et al., 2019) extends lifespan and improves neurobehavior in animal models, and in humans the donor’s age imprints the epigenetic age of reconstituted blood, suggesting a feasible route to immune system reset in older individuals. Overall, a personalized combination of p38/oxidative control, metabolic and autophagy correction, telomere maintenance, cdc42 inhibition, and—when appropriate—transplantation is recommended, coupled with rigorous oncologic surveillance and long-term follow-up.

### 3.2 T and B cell

Interventions for T-cell aging in older adults can be advanced through an integrated framework of “rebuilding numbers, preserving diversity, restoring function, and selectively de-senescing.” First, reconstituting the IL-7/IL-7R axis to expand the peripheral T-cell pool is supported by clinical data in HIV cohorts showing recombinant human IL-7 increases and activates both CD4^+^ and CD8^+^ T cells, providing a rationale for physiological recalibration in healthy elders. Second, limiting TCR repertoire contraction and clonal expansions—via pathogen management (e.g., CMV) and optimized, precision vaccination—helps maintain the naïve pool and antigen-recognition breadth. Third, reversing maladaptive phenotypic remodeling by inhibiting the sestrin–p38 stress pathway can prevent the age-associated shift of CD8^+^ T cells from TCR-dependent to NK-like activity, thereby restoring antigen specificity and enhancing vaccine responsiveness. Fourth, phenotype-guided, selective removal or reprogramming of “senescence-like” subsets (Yoshida et al., 2020) (CD28 low/−, CD27^−^, CD57^+^, CD45RA^+^ CD27^−^) is feasible; notably, a CD153-targeted peptide vaccine safely reduces CD153^+^ senescent T cells in mice and improves metabolic homeostasis, illustrating precise depletion without compromising normal immunity. Fifth (Lanna et al., 2017; Martínez and Blasco, 2017),correcting cellular senescence at its roots through telomere/telomerase-directed strategies—including indirect telomerase upregulation via the sestrin–p38 axis—may mitigate DNA damage and pro-inflammatory phenotypes while restoring proliferative capacity and durability. Overall, stratification by immune phenotype and combination regimens that couple IL-7 supplementation (Fry and Mackall, 2005; Rosenberg et al., 2006), sestrin–p38 inhibition, telomere maintenance, and targeted vaccination are recommended, with continuous monitoring for infection risk, hyperactivation or autoimmunity, and metabolic adverse effects to maximize the quantity, quality, and specificity of adaptive immunity in older individuals.

Therapeutic strategies for B-cell aging can follow the principles of “deplete age-promoting subsets, preserve beneficial populations, and rebuild function.” First, selectively remove age-associated B cells (Camell et al., 2019) (ABCs)—which are tightly linked to immunosenescence and inflammaging—by blocking the TLR7/9–IL-21/IFN-γ axis required for their differentiation or by employing targeted depletion approaches, while sparing the normal B-cell compartment. Second, target aged adipose B cells (AABs), which express high levels of IL-1R and are induced by IL-1β/IL-18: anti-CD20 antibodies or an IL-1 receptor antagonist (IL-1RA) can reduce AAB abundance and improve lipolysis and metabolic profiles in mice. Third, maintain and restore the antimicrobial “housekeeping” function of B-1 cells and their IgM secretion to counteract age-related contraction of the IgM repertoire and affinity, thereby strengthening primary responses to novel pathogens and vaccines. When necessary, a short-term, global B-cell “reset” using a CD19/B220/CD22 depletion cocktail may reactivate bone-marrow B lymphopoiesis and rejuvenate the peripheral pool; however, this strategy alone has not restored immune competence or vaccine responsiveness and should be combined with the above selective depletion and functional reconstruction measures. Integration with precision vaccination and individualized monitoring is recommended to enhance protection while managing infection risk and potential immunosuppression. Larger, well-controlled studies are needed to establish long-term safety and efficacy (Keren et al., 2011).

### 3.3 Macrophage

Framed around the triad of “restoring sensing—rebalancing polarization—promoting resolution,” the strategy can be summarized as follows (Jackaman et al., 2013): (i) Immune reprogramming: In aged mice, targeted IL-2/anti-CD40 combination therapy can re-enable macrophage capacity to activate T cells and to initiate M1-like inflammatory programs, indicating that functional reinstatement via co-stimulatory/cytokine axes is feasible. (ii) Metabolic and autophagy targeting: Aged macrophages exhibit reduced autophagy, dysregulated nutrient sensing, and mitochondrial dysfunction. Caloric restriction and its mimetics (Fabbiano et al., 2016; Fahy et al., 2019) (e.g., metformin, resveratrol, rapamycin) may improve energy metabolism and autophagy, modulate mTOR/AMPK pathways, correct M1/M2 imbalance, and dampen chronic inflammation, thereby potentially extending healthspan and enhancing innate immune competence. (iii) Pro-resolution enhancement: Delayed inflammatory resolution in older adults is linked to hyperactive p38 signaling and aberrant TIM-4 expression (De Maeyer et al., 2020). Oral p38 inhibition can normalize TIM-4-mediated efferocytosis and the resolution program and, by curbing CCL2-dependent recruitment of inflammatory monocytes, reduce local inflammation while augmenting antigen-specific responses. This approach is a promising option for age-related inflammatory diseases, though larger studies are required to establish efficacy and safety. (iv) Foundational functional support: In conjunction with the above, measures to counter diminished TLR expression and insufficient MHC-II induction should be implemented to bolster pathogen recognition and antigen presentation as the bedrock of a comprehensive regimen.

Overall, we recommend a core framework of “metabolic correction - precise pathway modulation - resolution promotion,” applied in individualized combinations, with ongoing monitoring for infection risk, potential immunosuppression, and metabolic adverse effects.

### 3.4 Other

Immunogeroprotective strategies can be grouped into four categories. (i) (Sun et al., 2010) Thymic regeneration: Centered on FOXN1, approaches include gene therapy (Oh et al., 2020), transplantation of FOXN1-high thymic epithelial stem cells (Campinoti et al., 2020), somatic cell reprogramming to generate ectopic thymus, and decellularized scaffolds repopulated with human cells to restore thymic architecture and naïve T-cell output; adjunctive factors such as KGF, IGF-1, BMP4, and IL-7/IL-22 may further enhance function (Chu et al., 2008; Min et al., 2007; Wertheimer et al., 2018). Because benefits may be offset by fibrosis of secondary lymphoid organs, antifibrotic or senolytic agents—or synthetic lymph nodes to remodel the microenvironment—should be considered in combination. (ii) Targeting immune metabolism: Caloric restriction (CR) and its mimetics attenuate inflammaging by inhibiting mTOR, activating AMPK/autophagy and sirtuins, and improving T and NK-cell function. Representative agents include metformin (which improves mitochondrial function and autophagy in aged CD4^+^ T cells; broad epidemiologic benefits; TAME in progress) (Cabreiro et al., 2013; Partridge et al., 2020), rapamycin (Strong et al., 2016) and other mTORC1 inhibitors (enhanced influenza vaccine responses, though phase III results have been mixed), resveratrol, spermidine, and α-ketoglutarate; infection risk and population-specific evidence must be balanced. (iii) Senescent-cell clearance (Xu et al., 2018): Senolytics that target anti-apoptotic pathways or β-galactosidase-activated prodrugs reduce the SASP and systemic inflammation (Amor et al., 2020); CAR-T cells engineered against markers such as uPAR offer targeted elimination. (iv) Epigenetic modulation: Interventions addressing aberrant DNA methylation (Garg et al., 2014) and chromatin states, suppressing retrotransposon activation (De Cecco et al., 2019; Simon et al., 2019) (e.g., via SIRT6 activation), and exploring DNMT/HDAC-directed agents may mitigate immunosenescence.

Overall, a combinatorial, stratified, and evidence-based program—integrating organ regeneration, metabolic reprogramming, senescent-cell clearance, and epigenetic modulation—is recommended, with vigilant monitoring for immune tolerance, tumor immunosurveillance trade-offs, and metabolic adverse effects, and with dedicated trials in healthy older adults versus disease cohorts plus long-term follow-up. The pathogenic mechanism and the corresponding treatment methods are shown in Table 1.

**TABLE 1:** Immunosenescence → Inflammaging → Coronary Artery Disease: Mechanisms and Targeted Interventions. (* Telomere-targeted interventions require strict oncologic monitoring).

Module	Aging shift	CAD effect	Key interventions (ultra-brief)
T cells	Naïve ↓; CD28^−^CD57^+^ ↑; TCR diversity ↓	Endothelial injury; inflammation/ROS ↑ → atherogenesis	IL-7 axis; CMV control + precision vaccines; sestrin–p38 block; selective depletion (e.g., CD153); cautious telomere support
B cells	FO IgG to OSEs ↑; MZ/B1 IgM ↓	IgG fuels plaques; IgM clears oxLDL/apoptotic cells	Deplete ABCs/AABs; rebuild B-1/natural IgM; IL-1RA or anti-CD20
Macrophages	Phagocytosis/TLR/MHC-II ↓; M1/M2 imbalance; SASP/ROS ↑	Chronic inflammation; poor resolution; thicker neointima	IL-2/anti-CD40 reprogramming; CR mimetics (metformin/resveratrol/rapamycin); oral p38 inhibition; boost TLR/MHC-II
Inflammaging	Persistent cytokines; DAMP–PRR; ROS/RNS ↑; CMV imprint	Endothelial damage; VSMC proliferation; foam cells; unstable plaques	CR/antioxidants; senolytics; vaccine/pathogen management
HSCs	DNA damage/ROS; telomere attrition; cdc42 ↑	Myeloid bias; lymphoid output ↓	p38 + antioxidants; Sirt3/autophagy (CMA) restore; telomere maintenance*; cdc42 inhibition (CASIN); young-donor grafts
System-level	Thymic involution; metabolic/epigenetic drift; senescent burden ↑	Immune regeneration ↓; inflammaging ↑	FOXN1-based thymic regeneration; CR/metformin/rapamycin/spermidine/α-KG; senolytics (β-gal prodrugs, uPAR CAR-T); SIRT6/DNMT/HDAC modulation

## 4 Conclusion and outlook

Coronary artery disease (CAD) is closely linked to immunosenescence, whereby age-related decline in immune function promotes chronic inflammation, vascular injury, and atherosclerosis. Through dysfunction of multiple immune cell populations—including T cells, B cells, and macrophages—immunosenescence attenuates immune responsiveness and sustains persistent inflammatory activity. In recent years, several intervention strategies targeting immunosenescence have shown preliminary promise, including restoration of hematopoietic stem-cell function, activation and rejuvenation of T- and B-cell compartments, modulation of macrophage activity, thymic regeneration, and immunometabolic reprogramming; together, these approaches offer new therapeutic directions for CAD. Future research should prioritize elucidating the mechanistic roles of immunosenescence in CAD, developing precise, personalized treatment strategies, and validating their safety and efficacy in large-scale clinical trials. Combination regimens that integrate immune modulation with metabolic interventions are likely to represent an important therapeutic avenue, with the potential to improve treatment efficacy and long-term patient outcomes.

## References

[B1] Allsopp R. C. Morin G. B. DePinho R. Harley C. B. Weissman I. L. (2003). Telomerase is required to slow telomere shortening and extend replicative lifespan of HSCs during serial transplantation. Blood 102 (2), 517–520. 10.1182/blood-2002-07-2334 12663456

[B2] Álvarez-Álvarez M. M. Zanetti D. Carreras-Torres R. Moral P. Athanasiadis G. (2017). A survey of sub-saharan gene flow into the mediterranean at risk loci for coronary artery disease. Eur. J. Hum. Genet. 25 (4), 472–476. 10.1038/ejhg.2016.200 28098150 PMC5386420

[B3] Álvarez-Heredia P. Reina-Alfonso I. Domínguez-Del-Castillo J. J. Gutiérrez-González C. Hassouneh F. Batista-Duharte A. (2023). Accelerated T-Cell immunosenescence in cytomegalovirus-seropositive individuals after severe acute respiratory syndrome coronavirus 2 infection. J. Infect. Dis. 228 (5), 576–585. 10.1093/infdis/jiad119 37103009 PMC10469128

[B4] Amor C. Feucht J. Leibold J. Ho Y. J. Zhu C. Alonso-Curbelo D. (2020). Senolytic CAR T cells reverse senescence-associated pathologies. Nature 583 (7814), 127–132. 10.1038/s41586-020-2403-9 32555459 PMC7583560

[B5] Basatemur G. L. Jørgensen H. F. Clarke M. C. H. Bennett M. R. Mallat Z. (2019). Vascular smooth muscle cells in atherosclerosis. Nat. Rev. Cardiol. 16 (12), 727–744. 10.1038/s41569-019-0227-9 31243391

[B6] Benjamin E. J. Blaha M. J. Chiuve S. E. Cushman M. Das S. R. Deo R. (2017). Heart disease and stroke Statistics-2017 update: a report from the American heart association. Circulation 135 (10), e146–e603. 10.1161/cir.0000000000000485 28122885 PMC5408160

[B7] Bergström I. Backteman K. Lundberg A. Ernerudh J. Jonasson L. (2012). Persistent accumulation of interferon-γ-producing CD8+CD56+ T cells in blood from patients with coronary artery disease. Atherosclerosis 224 (2), 515–520. 10.1016/j.atherosclerosis.2012.07.033 22882906

[B8] Binder C. J. Papac-Milicevic N. Witztum J. L. (2016). Innate sensing of oxidation-specific epitopes in health and disease. Nat. Rev. Immunol. 16 (8), 485–497. 10.1038/nri.2016.63 27346802 PMC7097710

[B9] Broadley I. Pera A. Morrow G. Davies K. A. Kern F. (2017). Expansions of cytotoxic CD4(+)CD28(-) T cells drive excess cardiovascular mortality in rheumatoid arthritis and other chronic inflammatory conditions and are triggered by CMV infection. Front. Immunol. 8, 195. 10.3389/fimmu.2017.00195 28303136 PMC5332470

[B10] Brown K. Xie S. Qiu X. Mohrin M. Shin J. Liu Y. (2013). SIRT3 reverses aging-associated degeneration. Cell Rep. 3 (2), 319–327. 10.1016/j.celrep.2013.01.005 23375372 PMC3582834

[B11] Cabreiro F. Au C. Leung K. Y. Vergara-Irigaray N. Cochemé H. M. Noori T. (2013). Metformin retards aging in *C. elegans* by altering microbial folate and methionine metabolism. Cell 153 (1), 228–239. 10.1016/j.cell.2013.02.035 23540700 PMC3898468

[B12] Camell C. D. Günther P. Lee A. Goldberg E. L. Spadaro O. Youm Y. H. (2019). Aging induces an Nlrp3 inflammasome-dependent expansion of adipose B cells that impairs metabolic homeostasis. Cell Metab. 30 (6), 1024–1039.e6. 10.1016/j.cmet.2019.10.006 31735593 PMC6944439

[B13] Campinoti S. Gjinovci A. Ragazzini R. Zanieri L. Ariza-McNaughton L. Catucci M. (2020). Reconstitution of a functional human thymus by postnatal stromal progenitor cells and natural whole-organ scaffolds. Nat. Commun. 11 (1), 6372. 10.1038/s41467-020-20082-7 33311516 PMC7732825

[B14] De Cecco M. Ito T. Petrashen A. P. Elias A. E. Skvir N. J. Criscione S. W. (2019). L1 drives IFN in senescent cells and promotes age-associated inflammation. Nature 566 (7742), 73–78. 10.1038/s41586-018-0784-9 30728521 PMC6519963

[B15] Centa M. Prokopec K. E. Garimella M. G. Habir K. Hofste L. Stark J. M. (2018). Acute loss of apolipoprotein E triggers an autoimmune response that accelerates atherosclerosis. Arterioscler. Thromb. Vasc. Biol. 38 (8), e145–e158. 10.1161/ATVBAHA.118.310802 29880490 PMC6173285

[B16] Chelvarajan R. L. Liu Y. Popa D. Getchell M. L. Getchell T. V. Stromberg A. J. (2006). Molecular basis of age-associated cytokine dysregulation in LPS-stimulated macrophages. J. Leukoc. Biol. 79 (6), 1314–1327. 10.1189/jlb.0106024 16603589

[B17] Chu Y. W. Schmitz S. Choudhury B. Telford W. Kapoor V. Garfield S. (2008). Exogenous insulin-like growth factor 1 enhances thymopoiesis predominantly through thymic epithelial cell expansion. Blood 112 (7), 2836–2846. 10.1182/blood-2008-04-149435 18658030 PMC2556619

[B18] Cui C. Y. Driscoll R. K. Piao Y. Chia C. W. Gorospe M. Ferrucci L. (2019). Skewed macrophage polarization in aging skeletal muscle. Aging Cell 18 (6), e13032. 10.1111/acel.13032 31478346 PMC6826159

[B19] Das M. M. Godoy M. Chen S. Moser V. A. Avalos P. Roxas K. M. (2019). Young bone marrow transplantation preserves learning and memory in old mice. Commun. Biol. 2, 73. 10.1038/s42003-019-0298-5 30820468 PMC6382867

[B20] Davila D. R. Edwards C. K. 3rd Arkins S. Simon J. Kelley K. W. (1990). Interferon-gamma-induced priming for secretion of superoxide anion and tumor necrosis factor-alpha declines in macrophages from aged rats. Faseb J. 4 (11), 2906–2911. 10.1096/fasebj.4.11.2165948 2165948

[B21] Ding A. Hwang S. Schwab R. (1994). Effect of aging on murine macrophages. Diminished response to IFN-gamma for enhanced oxidative metabolism. J. Immunol. 153 (5), 2146–2152. 10.4049/jimmunol.153.5.2146 7519641

[B22] Donato A. J. Morgan R. G. Walker A. E. Lesniewski L. A. (2015). Cellular and molecular biology of aging endothelial cells. J. Mol. Cell Cardiol. 89 (Pt B), 122–135. 10.1016/j.yjmcc.2015.01.021 25655936 PMC4522407

[B23] Dong S. Wang Q. Kao Y.-R. Diaz A. Tasset I. Kaushik S. (2021). Chaperone-mediated autophagy sustains haematopoietic stem-cell function. Nature 591 (7848), 117–123. 10.1038/s41586-020-03129-z 33442062 PMC8428053

[B24] Douna H. Amersfoort J. Schaftenaar F. H. Kroon S. van Puijvelde G. H. M. Kuiper J. (2019). Bidirectional effects of IL-10(+) regulatory B cells in Ldlr(-/-) mice. Atherosclerosis 280, 118–125. 10.1016/j.atherosclerosis.2018.11.019 30500604

[B25] Dumitriu I. E. Araguás E. T. Baboonian C. Kaski J. C. (2009). CD4+ CD28 null T cells in coronary artery disease: when helpers become killers. Cardiovasc Res. 81 (1), 11–19. 10.1093/cvr/cvn248 18818214

[B26] Fabbiano S. Suárez-Zamorano N. Rigo D. Veyrat-Durebex C. Stevanovic Dokic A. Colin D. J. (2016). Caloric restriction leads to browning of white adipose tissue through type 2 immune signaling. Cell Metab. 24 (3), 434–446. 10.1016/j.cmet.2016.07.023 27568549

[B27] Fahy G. M. Brooke R. T. Watson J. P. Good Z. Vasanawala S. S. Maecker H. (2019). Reversal of epigenetic aging and immunosenescent trends in humans. Aging Cell 18 (6), e13028. 10.1111/acel.13028 31496122 PMC6826138

[B28] Feldman N. Rotter-Maskowitz A. Okun E. (2015). DAMPs as mediators of sterile inflammation in aging-related pathologies. Ageing Res. Rev. 24 (Pt A), 29–39. 10.1016/j.arr.2015.01.003 25641058

[B29] Ford T. J. Corcoran D. Berry C. (2018). Stable coronary syndromes: pathophysiology, diagnostic advances and therapeutic need. Heart 104 (4), 284–292. 10.1136/heartjnl-2017-311446 29030424 PMC5861393

[B30] Formentini M. Navas A. Hassouneh F. Lopez-Sejas N. Alonso C. Tarazona R. (2021). Impact of cytomegalovirus and age on T-Cell subsets defined by CD161, CD300a, and/or CD57 expression in healthy andalusians. J. Gerontol. A Biol. Sci. Med. Sci. 76 (11), 1946–1953. 10.1093/gerona/glab140 33993242

[B31] Franceschi C. Campisi J. (2014). Chronic inflammation (inflammaging) and its potential contribution to age-associated diseases. J. Gerontol. A Biol. Sci. Med. Sci. 69 (Suppl. 1), S4–S9. 10.1093/gerona/glu057 24833586

[B32] Franceschi C. Garagnani P. Parini P. Giuliani C. Santoro A. (2018). Inflammaging: a new immune-metabolic viewpoint for age-related diseases. Nat. Rev. Endocrinol. 14 (10), 576–590. 10.1038/s41574-018-0059-4 30046148

[B33] Frangogiannis N. G. (2021). Cardiac fibrosis. Cardiovasc Res. 117 (6), 1450–1488. 10.1093/cvr/cvaa324 33135058 PMC8152700

[B34] Frodermann V. Nahrendorf M. (2018). Macrophages and cardiovascular health. Physiol. Rev. 98 (4), 2523–2569. 10.1152/physrev.00068.2017 30156496 PMC6442921

[B35] Fry T. J. Mackall C. L. (2005). The many faces of IL-7: from lymphopoiesis to peripheral T cell maintenance. J. Immunol. 174 (11), 6571–6576. 10.4049/jimmunol.174.11.6571 15905493

[B36] Garg S. K. Delaney C. Toubai T. Ghosh A. Reddy P. Banerjee R. (2014). Aging is associated with increased regulatory T-cell function. Aging Cell 13 (3), 441–448. 10.1111/acel.12191 24325345 PMC4032602

[B37] Ghamar Talepoor A. Doroudchi M. (2022). Immunosenescence in atherosclerosis: a role for chronic viral infections. Front. Immunol. 13, 945016. 10.3389/fimmu.2022.945016 36059478 PMC9428721

[B38] Global regional (2015). Global, regional, and national age-sex specific all-cause and cause-specific mortality for 240 causes of death, 1990-2013: a systematic analysis for the global burden of disease study 2013. Lancet 385 (9963), 117–171. 10.1016/S0140-6736(14)61682-2 25530442 PMC4340604

[B39] Goronzy J. J. Weyand C. M. (2013). Understanding immunosenescence to improve responses to vaccines. Nat. Immunol. 14 (5), 428–436. 10.1038/ni.2588 23598398 PMC4183346

[B40] Hammad H. Lambrecht B. N. (2008). Dendritic cells and epithelial cells: linking innate and adaptive immunity in asthma. Nat. Rev. Immunol. 8 (3), 193–204. 10.1038/nri2275 18301423

[B41] Herrero C. Marqués L. Lloberas J. Celada A. (2001). IFN-gamma-dependent transcription of MHC class II IA is impaired in macrophages from aged mice. J. Clin. Invest 107 (4), 485–493. 10.1172/JCI11696 11181648 PMC199261

[B42] Herrmann J. Kaski J. C. Lerman A. (2012). Coronary microvascular dysfunction in the clinical setting: from mystery to reality. Eur. Heart J. 33 (22), 2771–2782b. 10.1093/eurheartj/ehs246 22915165 PMC3498003

[B43] Hosokawa K. MacArthur B. D. Ikushima Y. M. Toyama H. Masuhiro Y. Hanazawa S. (2017). The telomere binding protein Pot1 maintains haematopoietic stem cell activity with age. Nat. Commun. 8 (1), 804. 10.1038/s41467-017-00935-4 28986560 PMC5630588

[B44] Hsieh C.-C. Papaconstantinou J. (2002). The effect of aging on p38 signaling pathway activity in the mouse liver and in response to ROS generated by 3-nitropropionic acid. Mech. ageing Dev. 123 (11), 1423–1435. 10.1016/s0047-6374(02)00084-2 12425949

[B45] Jackaman C. Radley-Crabb H. G. Soffe Z. Shavlakadze T. Grounds M. D. Nelson D. J. (2013). Targeting macrophages rescues age-related immune deficiencies in C57BL/6J geriatric mice. Aging Cell 12 (3), 345–357. 10.1111/acel.12062 23442123

[B46] Jonasson L. Tompa A. Wikby A. (2003). Expansion of peripheral CD8+ T cells in patients with coronary artery disease: relation to cytomegalovirus infection. J. Intern Med. 254 (5), 472–478. 10.1046/j.1365-2796.2003.01217.x 14535969

[B47] Keren Z. Naor S. Nussbaum S. Golan K. Itkin T. Sasaki Y. (2011). B-cell depletion reactivates B lymphopoiesis in the BM and rejuvenates the B lineage in aging. Blood 117 (11), 3104–3112. 10.1182/blood-2010-09-307983 21228330

[B48] Khera A. V. Kathiresan S. (2017). Genetics of coronary artery disease: discovery, biology and clinical translation. Nat. Rev. Genet. 18 (6), 331–344. 10.1038/nrg.2016.160 28286336 PMC5935119

[B49] Kovina M. V. Karnaukhov A. V. Krasheninnikov M. E. Gazheev S. T. Sergievich L. A. (2019). Extension of maximal lifespan and high bone marrow chimerism after nonmyeloablative syngeneic transplantation of bone marrow from young to old mice. Front. Genet. 10, 310. 10.3389/fgene.2019.00310 31031800 PMC6473025

[B50] Lanna A. Gomes D. C. Muller-Durovic B. McDonnell T. Escors D. Gilroy D. W. (2017). A sestrin-dependent Erk-Jnk-p38 MAPK activation complex inhibits immunity during aging. Nat. Immunol. 18 (3), 354–363. 10.1038/ni.3665 28114291 PMC5321575

[B51] Leon M. L. Zuckerman S. H. (2005). Gamma interferon: a central mediator in atherosclerosis. Inflamm. Res. 54 (10), 395–411. 10.1007/s00011-005-1377-2 16283107

[B52] Li Z. Li J. Bu X. Liu X. Tankersley C. G. Wang C. (2011). Age-induced augmentation of p38 MAPK phosphorylation in mouse lung. Exp. Gerontol. 46 (8), 694–702. 10.1016/j.exger.2011.04.005 21570457

[B53] Libby P. Ridker P. M. Hansson G. K. Leducq Transatlantic Network on Atherothrombosis (2009). Inflammation in atherosclerosis: from pathophysiology to practice. J. Am. Coll. Cardiol. 54 (23), 2129–2138. 10.1016/j.jacc.2009.09.009 19942084 PMC2834169

[B54] Linehan E. Dombrowski Y. Snoddy R. Fallon P. G. Kissenpfennig A. Fitzgerald D. C. (2014). Aging impairs peritoneal but not bone marrow-derived macrophage phagocytosis. Aging Cell 13 (4), 699–708. 10.1111/acel.12223 24813244 PMC4326936

[B55] Liu Z. Liang Q. Ren Y. Guo C. Ge X. Wang L. (2023). Immunosenescence: molecular mechanisms and diseases. Signal Transduct. Target Ther. 8 (1), 200. 10.1038/s41392-023-01451-2 37179335 PMC10182360

[B56] De Maeyer R. P. H. van de Merwe R. C. Louie R. Bracken O. V. Devine O. P. Goldstein D. R. (2020). Blocking elevated p38 MAPK restores efferocytosis and inflammatory resolution in the elderly. Nat. Immunol. 21 (6), 615–625. 10.1038/s41590-020-0646-0 32251403 PMC7983074

[B57] Mahbub S. Deburghgraeve C. R. Kovacs E. J. (2012). Advanced age impairs macrophage polarization. J. Interferon Cytokine Res. 32 (1), 18–26. 10.1089/jir.2011.0058 22175541 PMC3255514

[B58] Martínez P. Blasco M. A. (2017). Telomere-driven diseases and telomere-targeting therapies. J. Cell Biol. 216 (4), 875–887. 10.1083/jcb.201610111 28254828 PMC5379954

[B59] Mauro A. G. Bonaventura A. Mezzaroma E. Quader M. Toldo S. (2019). NLRP3 inflammasome in acute myocardial infarction. J. Cardiovasc. Pharmacol. 74 (3), 175–187. 10.1097/FJC.0000000000000717 31356555

[B60] Min D. Panoskaltsis-Mortari A. Kuro O. M. Holländer G. A. Blazar B. R. Weinberg K. I. (2007). Sustained thymopoiesis and improvement in functional immunity induced by exogenous KGF administration in murine models of aging. Blood 109 (6), 2529–2537. 10.1182/blood-2006-08-043794 17138819 PMC1852207

[B61] Namekawa T. Snyder M. R. Yen J. H. Goehring B. E. Leibson P. J. Weyand C. M. (2000). Killer cell activating receptors function as costimulatory molecules on CD4+CD28null T cells clonally expanded in rheumatoid arthritis. J. Immunol. 165 (2), 1138–1145. 10.4049/jimmunol.165.2.1138 10878393

[B62] National Heart L. Institute B. (2006). Incidence and prevalence: 2006 chart book on cardiovascular and lung diseases. Bethesda, MD: National Institutes of Health.

[B63] Nikolich-Žugich J. (2018). The twilight of immunity: emerging concepts in aging of the immune system. Nat. Immunol. 19 (1), 10–19. 10.1038/s41590-017-0006-x 29242543

[B64] Oh J. Wang W. Thomas R. Su D. M. (2020). Thymic rejuvenation *via* FOXN1-reprogrammed embryonic fibroblasts (FREFs) to counteract age-related inflammation. JCI Insight 5 (18), e140313. 10.1172/jci.insight.140313 32790650 PMC7526556

[B65] de Oliveira Laterza Ribeiro M. Correia V. M. Herling de Oliveira L. L. Soares P. R. Scudeler T. L. (2023). Evolving diagnostic and management advances in coronary heart disease. Life (Basel) 13 (4), 951. 10.3390/life13040951 37109480 PMC10143565

[B66] Partridge L. Fuentealba M. Kennedy B. K. (2020). The quest to slow ageing through drug discovery. Nat. Rev. Drug Discov. 19 (8), 513–532. 10.1038/s41573-020-0067-7 32467649

[B67] Rosenberg S. A. Sportès C. Ahmadzadeh M. Fry T. J. Ngo L. T. Schwarz S. L. (2006). IL-7 administration to humans leads to expansion of CD8+ and CD4+ cells but a relative decrease of CD4+ T-regulatory cells. J. Immunother. 29 (3), 313–319. 10.1097/01.cji.0000210386.55951.c2 16699374 PMC1473976

[B68] Ross R. (1999). Atherosclerosis--an inflammatory disease. N. Engl. J. Med. 340 (2), 115–126. 10.1056/NEJM199901143400207 9887164

[B69] Sage A. P. Nus M. Baker L. L. Finigan A. J. Masters L. M. Mallat Z. (2015). Regulatory B cell-specific interleukin-10 is dispensable for atherosclerosis development in mice. Arterioscler. Thromb. Vasc. Biol. 35 (8), 1770–1773. 10.1161/ATVBAHA.115.305568 26088575

[B70] Sage A. P. Tsiantoulas D. Binder C. J. Mallat Z. (2019). The role of B cells in atherosclerosis. Nat. Rev. Cardiol. 16 (3), 180–196. 10.1038/s41569-018-0106-9 30410107

[B72] Santoro A. Bientinesi E. Monti D. (2021). Immunosenescence and inflammaging in the aging process: age-related diseases or longevity? Ageing Res. Rev. 71, 101422. 10.1016/j.arr.2021.101422 34391943

[B73] Sharma R. (2021). Perspectives on the dynamic implications of cellular senescence and immunosenescence on macrophage aging biology. Biogerontology 22 (6), 571–587. 10.1007/s10522-021-09936-9 34490541

[B74] Sharma R. Kapila R. Haq M. R. Salingati V. Kapasiya M. Kapila S. (2014). Age-associated aberrations in mouse cellular and humoral immune responses. Aging Clin. Exp. Res. 26 (4), 353–362. 10.1007/s40520-013-0190-y 24343854

[B75] Simon M. Van Meter M. Ablaeva J. Ke Z. Gonzalez R. S. Taguchi T. (2019). LINE1 derepression in aged wild-type and SIRT6-Deficient mice drives inflammation. Cell Metab. 29 (4), 871–885.e5. 10.1016/j.cmet.2019.02.014 30853213 PMC6449196

[B76] Snijckers R. P. M. Foks A. C. (2024). Adaptive immunity and atherosclerosis: aging at its crossroads. Front. Immunol. 15, 1350471. 10.3389/fimmu.2024.1350471 38686373 PMC11056569

[B77] Soehnlein O. Libby P. (2021). Targeting inflammation in atherosclerosis - from experimental insights to the clinic. Nat. Rev. Drug Discov. 20 (8), 589–610. 10.1038/s41573-021-00198-1 33976384 PMC8112476

[B78] Strong R. Miller R. A. Antebi A. Astle C. M. Bogue M. Denzel M. S. (2016). Longer lifespan in male mice treated with a weakly estrogenic agonist, an antioxidant, an α-glucosidase inhibitor or a Nrf2-inducer. Aging Cell 15 (5), 872–884. 10.1111/acel.12496 27312235 PMC5013015

[B79] Sun L. Guo J. Brown R. Amagai T. Zhao Y. Su D. M. (2010). Declining expression of a single epithelial cell-autonomous gene accelerates age-related thymic involution. Aging Cell 9 (3), 347–357. 10.1111/j.1474-9726.2010.00559.x 20156205 PMC2894280

[B80] Swift M. E. Burns A. L. Gray K. L. DiPietro L. A. (2001). Age-related alterations in the inflammatory response to dermal injury. J. Invest Dermatol. 117 (5), 1027–1035. 10.1046/j.0022-202x.2001.01539.x 11710909

[B81] Takx R. A. Blomberg B. A. El A. H. Habets J. de Jong P. A. Nagel E. (2015). Diagnostic accuracy of stress myocardial perfusion imaging compared to invasive coronary angiography with fractional flow reserve meta-analysis. Circ. Cardiovasc Imaging 8 (1), e002666. 10.1161/CIRCIMAGING.114.002666 25596143

[B82] Téo F. H. de Oliveira R. T. Mamoni R. L. Ferreira M. C. S. Nadruz W. Jr Coelho O. R. (2013). Characterization of CD4+CD28null T cells in patients with coronary artery disease and individuals with risk factors for atherosclerosis. Cell Immunol. 281 (1), 11–19. 10.1016/j.cellimm.2013.01.007 23416719

[B83] Tsao C. W. Aday A. W. Almarzooq Z. I. Alonso A. Beaton A. Z. Bittencourt M. S. (2022). Heart disease and stroke Statistics-2022 update: a report from the American heart association. Circulation 145 (8), e153–e639. 10.1161/CIR.0000000000001052 35078371

[B84] Tsiantoulas D. Gruber S. Binder C. J. (2012). B-1 cell immunoglobulin directed against oxidation-specific epitopes. Front. Immunol. 3, 415. 10.3389/fimmu.2012.00415 23316200 PMC3540410

[B85] Ucar D. Márquez E. J. Chung C. H. Marches R. Rossi R. J. Uyar A. (2017). The chromatin accessibility signature of human immune aging stems from CD8(+) T cells. J. Exp. Med. 214 (10), 3123–3144. 10.1084/jem.20170416 28904110 PMC5626401

[B86] Vallejo A. N. Brandes J. C. Weyand C. M. Goronzy J. J. (1999). Modulation of CD28 expression: distinct regulatory pathways during activation and replicative senescence. J. Immunol. 162 (11), 6572–6579. 10.4049/jimmunol.162.11.6572 10352273

[B87] Wertheimer T. Velardi E. Tsai J. Cooper K. Xiao S. Kloss C. C. (2018). Production of BMP4 by endothelial cells is crucial for endogenous thymic regeneration. Sci. Immunol. 3 (19), eaal2736. 10.1126/sciimmunol.aal2736 29330161 PMC5795617

[B88] Wong C. K. Smith C. A. Sakamoto K. Kaminski N. Koff J. L. Goldstein D. R. (2017). Aging impairs alveolar macrophage phagocytosis and increases influenza-induced mortality in mice. J. Immunol. 199 (3), 1060–1068. 10.4049/jimmunol.1700397 28646038 PMC5557035

[B89] Wynn T. A. Chawla A. Pollard J. W. (2013). Macrophage biology in development, homeostasis and disease. Nature 496 (7446), 445–455. 10.1038/nature12034 23619691 PMC3725458

[B90] Xu M. Pirtskhalava T. Farr J. N. Weigand B. M. Palmer A. K. Weivoda M. M. (2018). Senolytics improve physical function and increase lifespan in old age. Nat. Med. 24 (8), 1246–1256. 10.1038/s41591-018-0092-9 29988130 PMC6082705

[B91] Yoshida S. Nakagami H. Hayashi H. Ikeda Y. Sun J. Tenma A. (2020). The CD153 vaccine is a senotherapeutic option for preventing the accumulation of senescent T cells in mice. Nat. Commun. 11 (1), 2482. 10.1038/s41467-020-16347-w 32424156 PMC7235045

[B92] Yusuf S. Hawken S. Ounpuu S. Dans T. Avezum A. Lanas F. (2004). Effect of potentially modifiable risk factors associated with myocardial infarction in 52 countries (the INTERHEART study): case-control study. Lancet 364 (9438), 937–952. 10.1016/S0140-6736(04)17018-9 15364185

[B93] Zandi S. Nakao S. Chun K. H. Fiorina P. Sun D. Arita R. (2015). ROCK-isoform-specific polarization of macrophages associated with age-related macular degeneration. Cell Rep. 10 (7), 1173–1186. 10.1016/j.celrep.2015.01.050 25704819 PMC5219927

